# Selective nanosensor based on folic acid imprinted nanostructures

**DOI:** 10.55730/1300-0527.3428

**Published:** 2022-04-11

**Authors:** Kevser KUŞAT, Serdar ŞANLI, Suna TİMUR, Sinan AKGÖL

**Affiliations:** 1 Department of Chemistry, Faculty of Science, Dokuz Eylül University, İzmir, Turkey; 2 Department of Chemistry, Faculty of Science & Arts, Ordu University, Ordu, Turkey; 3 Department of Biochemistry, Faculty of Science, Ege University, İzmir, Turkey

**Keywords:** Folic acid, nanostructures, MIP, nanobiosensors

## Abstract

Folic acid, which provides the transfer of single carbon atoms in synthesis reactions and metabolic cycles in metabolism, is very important for metabolism. Folic acid also plays an important role in nucleotide synthesis and methylation reactions. There are many disorders caused by defective folic acid metabolism and lack of folic acid. Today, innovative, cost-effective methods are needed to develop folic acid determination methods. The main objective of this study is the development of surface-printed carbon electrodes (SPCE) modified with folic acid imprinted nanostructures (FA-Imp-poly(MPTS-rGO-co-NAT), which will be used for the first time for folic acid determination in commercially human blood serum. For this purpose, the synthesis of nanostructures has been carried out and characterized by FTIR, SEM-EDS, and AFM. Then, a new chemically modified nanosensor was fabricated for the determination of folic acid using folic acid imprinted nanostructures. Differential pulse voltammetry (DPV) and circular voltammetry (CV) methods were used as electrochemical methods in the FA-imprinted-nanosensor studies. Measurements in differential pulse voltammetry were performed at an application speed of 0.005 volts per second in the potential range of −0.4 to 0.6 volts. As a result of the circular voltammetric method, an idea about the surface was obtained with the voltammograms obtained. The detection limit (LOD) of the developed FA-imprinted-nanosensor was 7.54 ng/mL and the determination limit (LOQ) was 25.14 ng/mL. FA analytical (10 and 20 ng/mL) was added to commercial synthetic serum samples by the standard adding method and RSD values of 0.092% and 0.734% were found in the DPV technique and measurements respectively. This manuscript demonstrated a novel, simple, selective, and rapid FA-imprinted-nanosensor for determining the FA in the biological samples.

## 1. Introduction

Folic acid metabolism plays an important role in the de-novo synthesis of purines and thymedate, which are necessary for DNA replication and repair. Therefore, errors or deficiencies in the distribution of methyl groups due to folate metabolism affect both methylation and DNA synthesis processes that play an important role in cancer development [1]. Defect in folate metabolism also causes cardiovascular diseases, neural tube defects, cleft lip and palate, late pregnancy complications, and neurodegenerative diseases [2]. It has also been reported that folic acid deficiency is associated with congenital malformations of the spine, skull and brain in infant development during pregnancy [3]. Therefore, the sensitive determination of folic acid in the clinical site is very important in diagnosing many diseases. Sensor systems have recently become the most used and intended devices in clinical field diagnosis and diagnosis methods due to their fast response time and low-cost features. These devices offer many advantages such as the ability to analyze small volume samples, faster analysis time, compliance with automation procedures, and increased reliability and repeatability [4].

Electrochemical determination methods are fast, inexpensive, and easy to apply, which allows accurate measurement of the quantities of various compounds in a wide range of samples [5]. Previously, electrochemical analyses of pharmaceutical compounds were carried out using mercury-based electrodes that replaced graphite and metallic macro electrodes [6]. Today, macro-electrodes are produced from pyrolytic graphene, glassy carbon or boron diamond electrodes that can also be used by modifying or modifying with micro and nanostructures [7]. These developments have revealed new types of biosensors based on surface-printed electrodes (SPE) for the determination of various compounds in a wide range of sample matrixes. SPE’s are disposable devices with plenty of uses in a wide range of fields, including analytical chemistry, drug control, and clinical and environmental analysis due to significant developments in the last decade [8]. SPE-based biosensors can be considered as tools that provide important advantages such as low design limit, simple operation, low cost, portability and miniaturization potential, analytical detection of pharmaceuticals in a wide range of samples [9].

Molecular imprinting is an important method based on the use of materials capable of recognizing the target molecule specifically. Molecularly imprinted materials (MIPs) have many advantages such as selectivity, stability, reusability, and cost-effectiveness. However, MIPs prepared with the traditional technique have some disadvantages such as missing template removal, low binding capacity, high diffusion barrier, and low area accessibility. Because most of the imprinted areas are in the interior of the MIP material, in order to solve these problems, a surface imprinting technique has been developed in recent years, which involves the creation of the MIP layer on the surface of nanostructures. This method provides the advantages of higher bonding capacity and faster bonding kinetics on the material surface [10]. Due to the properties of MIPs combined with electrochemical studies, such as ease of use and low cost, their applications in the field of sensors have also increased today [11]. However, before MIP-based sensors take part in the sensor market, some problems still need to be overcome. The most important of these problems is the change in the distance of the imprinted cavities to the sensor surface and consequently the low signaling [12]. To prevent this problem and improve electrochemical signaling, the researchers focused on developing ultra-thin and homogeneous polymeric films on the surface of nano-sized support material with outstanding electrical conductivity such as graphene and an extremely large surface area. This ensures greater selectivity for thin MIP layers [13].

Since graphene has a wider specific surface area than 3D silica and other nanoparticles, it is quite suitable for supporting MIP to be produced by the surface imprinting technique. Located on the surface of graphene (graphene/MIP composite), MIP not only exhibits faster adsorption and desorption dynamics but also demonstrates a high binding capacity and high selectivity toward the target molecule [14]. In addition, graphene, which has a large π-electron system, has a strong affinity against carbon-based ring structures found in drugs, pollutants, and biomolecules [15]. In addition to all this, graphene is highly suitable to produce high-precision electrochemical MIP sensors thanks to its impressive properties such as high electron transfer speed, high heat, and electrical conductivity, exceptional elasticity and hardness, and high thermal stability [16]. Graphene has two degrees of oxidation; graphene oxide (GO) and reduced graphene oxide (rGO). GO has low conductivity and is water-soluble, while rGO has good conductivity and poor water solubility. The excellent solubility of GO in an aqueous solution is mainly due to oxygen-rich hydrophilic groups such as hydroxyl, epoxide, and carboxylic groups. These functional groups on the surface can interact with functional groups of the molecule that is intended to bind to the surface, providing a large number of reaction zones [17]. In addition, GO increases the detection area per unit volume due to its wide specific surface area (2630 m^2^g^−^^1^), which provides high sensitivity for the sensor device [18]. However, as mentioned above, GO’s hosting of oxygen-rich functional groups causes the electrical conductivity to decrease. Therefore, especially in sensor applications, GO is reduced. After the reduction, most oxygen-containing groups in GO, especially hydroxyl, epoxide, and carboxyl groups, are completely removed and converted into graphene rich in π-conjugation, i.e. reduced graphene oxide [19]. π-conjugation in graphene layers restores the conductivity of graphene. But it reduces its solubility in water and many other organic solvents. In addition, rGO is not very compatible with some other materials, such as polymer matrices. Various techniques have been developed to solve this problem, including the physical adsorbing of functional molecules to graphene layers and the chemical binding of functional groups to the graphene surface [20]. To date, the distribution of rGO in aqueous solutions has been achieved by physically adsorbing molecules and polymers containing functional groups that dissolve in water into rGO layers. But the presence of such stabilizers is undesirable for most applications [19]. Although there are many studies on surface-imprinting composites obtained using GO, studies on surface-imprinted composites with rGO are very few, especially those that are intended to be produced in the desired nature due to the poor dispersion of graphene layers in various solvents.

In this study, in the first step, folic acid was molecularly imprinted in the presence of silanized rGO (MPTS-rGO), using a functional comonomer (N-[tris(hydroxymethyl)methyl] acrylamide (NAT)), cross-linker and initiator molecules. This synthesized nanostructure [(FA-Imp-poly(MPTS-rGO-co-NAT)] was characterized by FTIR, SEM-EDS, AFM, and Elemental analysis methods. In the second step, a surface-imprinted carbon electrode (SPCE) was developed using (FA-Imp-poly(MPTS-rGO-co-NAT) nanostructure. The surface imprinted carbon electrode was modified, and measurements were made with ascorbic acid, dopamine selectivity studies, which are structural analogues of folic acid, and electrochemical studies with circular voltammetry (CV) and differential pulse voltammetry (DPV). Analytical characteristics of the developed nanosensor system (standard deviation, observance limit, determination limit, etc.) were determined. In this way, the analytical performance of the developed nanosensor system was evaluated. Kinetic analyses of the developed nanosensor system were carried out with commercial blood samples.

## 2. Materials and methods

In this study, graphite powder, potassium permanganate, H_2_SO_4_, H_3_PO_4_, HCI, NAT (N-(Hydroxymethyl) Methyl) Acrylamide), MPTS (3-(Trimethoxysilyl) Propyl Methacrylate), ethylene glycol dimethacrylate (EGDMA), potassium persulate (KPS), commercial blood serum (Human Blood/SIGMA ALDRICH BCR635-1EA) was used. All chemicals are purchased from Sigma-Aldrich. DRP-C1,10-U75 Curtain Printed Carbon Electrode and Connector for DRP-CAST Curtain Printed Electrode are used.

### 2.1. Synthesis of (FA-Imp-poly(MPTS-rGO-co-NAT) nanostructures

The first step of synthesis of (FA-Imp-poly(MPTS-rGO-co-NAT) nanostructures is graphene oxide synthesis by the Improved Hummers method. Then comes the silanization (MPTS-GO) and reduction (MPTS-rGO) steps of graphene oxide. Finally, folic acid imprinted polymeric nanostructures (FA-Imp-poly(MPTS-rGO-co-NAT) were synthesized by free radical polymerization method. Folic acid was used as a template molecule for the synthesis of folic acid-imprints nanostructures [FA-Imp-rGO-co-NAT), N-(Hydroxymethyl) Methyl) Acrylamide, (NAT) as a functional monomer, ethylene glycol dimethacrylate (EGDMA) as a cross-linker, and potassium persulfate (KPS) as the initiator in this method.

#### 2.1.1. Synthesis of graphene oxide by Improved Hummers method

**Figure 1:**
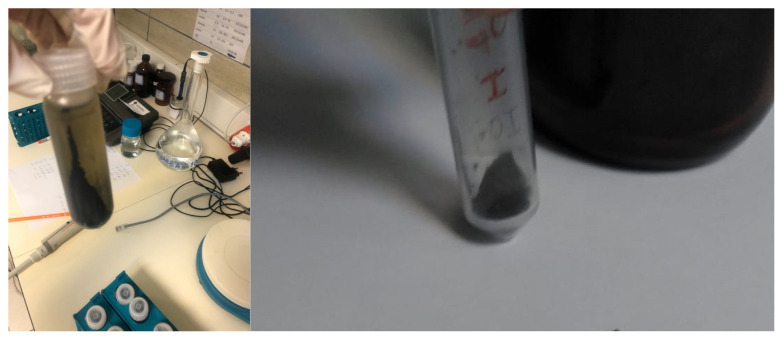
Photographs for synthesized graphene oxide (GO).

Graphene oxide was prepared from graphene powder with improved Hummer’s method [21]. In summary, 2 g of graphite powder, 12 g of potassium permanganate, and 261 mL of acid (235 mL H_2_SO_4_ + 26 mL H_3_PO_4_) were stirred at 50 °C in a balloon reactor for 12 h. This mixture was then poured over the mixture containing 260 mL of pure water and 2 mL of 30% H_2_O_2_, which was frozen. Then the final solution obtained was centrifuged for 1 h at 18,000 rpm. The precipitate was taken and dried overnight at 80 °C, first with a 10% HCI solution and then by washing with ethanol (Figure 1).

#### 2.1.2. Silanization of graphene oxide (MPTS-GO)

For silanization, 0.35 g of GO was added to the three-neck balloon containing 200 mL ethanol and 100 mL of water and sonicated by ultrasonication for 60 min. MPTS (1 mL) was added to 15 mL of water and kept the pH value between 4–5 with acetic acid to be fully hydrolyzed and added to the graphene oxide solution and stirred for silanization at 2 h 60–65 °C. MPTS-GO was filtered and repeatedly washed with methanol and water.

#### 2.1.3. Reduction of MPTS-GO

The reduction was carried out with ascorbic acid, thus obtaining reduced silanized graphene oxide (MPTS-rGO). Homogeneous dispersion of MPTS-GO (5.0 mL) was added to 5.0 g of ascorbic acid and stirred for 30 min at 60 °C. Then the last resulting solution was precipitated for 30 min at 4000 rpm. H_2_0_2_ (30%) was added to the precipitated substance and ascorbic acid that did not react was removed. The resulting black product was centrifuged and washed three times with ethanol and pure water, then dried overnight at 80 °C [22].

#### 2.1.4. Synthesis of folic acid imprinted polymeric nanostructures [(FA-Imp-poly(MPTS-rGO-co-NAT)]

As we mentioned before, folic acid was used as a template molecule for the synthesis of folic acid-imprints nanostructures [FA-Imp-rGO-co-NAT), NAT as a functional monomer, ethylene glycol dimethacrylate (EGDMA) as a cross-linker, and potassium persulphate (KPS) as the initiator (Table 1).

Functional monomer (NAT), which determined the optimum mole ratio with template molecule FA, was subjected to 2 h in 5 mL dimethylformamide (DMF). The template molecule (FA) and functional monomer (NAT) were pretreated for 2 h. MPTS-rGO nanostructures were dispersed in 20 mL of DMF then mixed with the precomplex solution and passed through nitrogen (N_2_) gas for 3 min.

Molecular imprinting was performed with free radical polymerization method for 24 h at 60 °C in the presence of EGDMA as a crosslinker and 0.02 g of KPS as an initiator. Polymerization conditions and the recipe for polymerization conditions are given in Table 1. EGDMA was used as 10 times the ratio of functional monomer moles. Because the cross-binding ratio in molecular imprinting is many times higher than the total amount of monomer moles, it plays a role in the stability of the imprinted regions belonging to the target molecule, and if this mole ratio is low, the imprinting zones are close to each other, and the imprinting efficiency decreases. In addition, the amount of potassium persulphate (KPS) selected as the initiator is taken as a reference from the study conducted by Kuru et al. (2017) and Karakoç et al. (2009) [23,24]. After the resulting folic acid-imprinted nanostructures were precipitated at 18,000 rpm for 1 h, monomer, substance, and initiator that did not react by washing once with deionized water, once with ethyl alcohol, and again with deionized water were removed. After washing, folic acid, which is the template molecule, was removed from the main structure using a desorption solution consisting of acetic acid:methanol (1:9) mixture [25].

**Figure 2:**
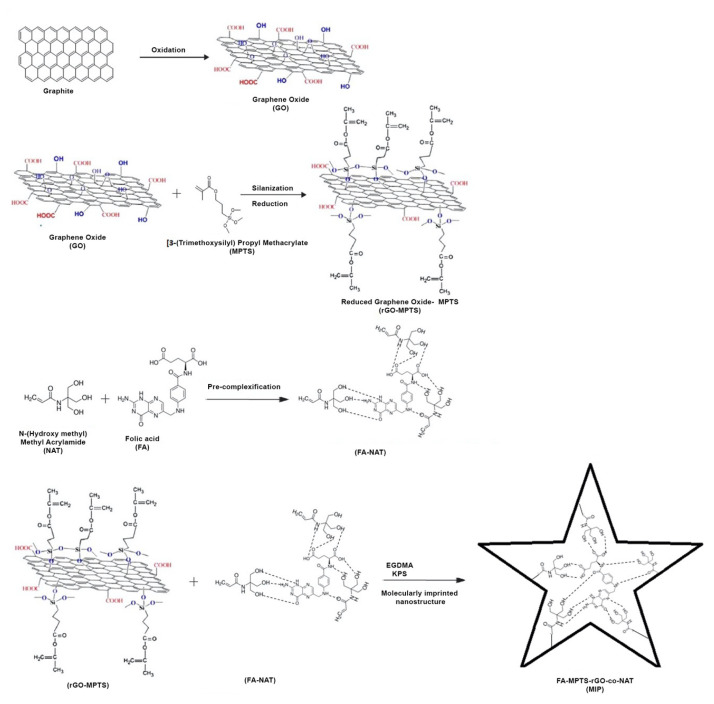
Synthesis steps and possible chemical interactions of FA-Imp-poly(MPTS-rGO-co-NAT).

**Figure 3:**
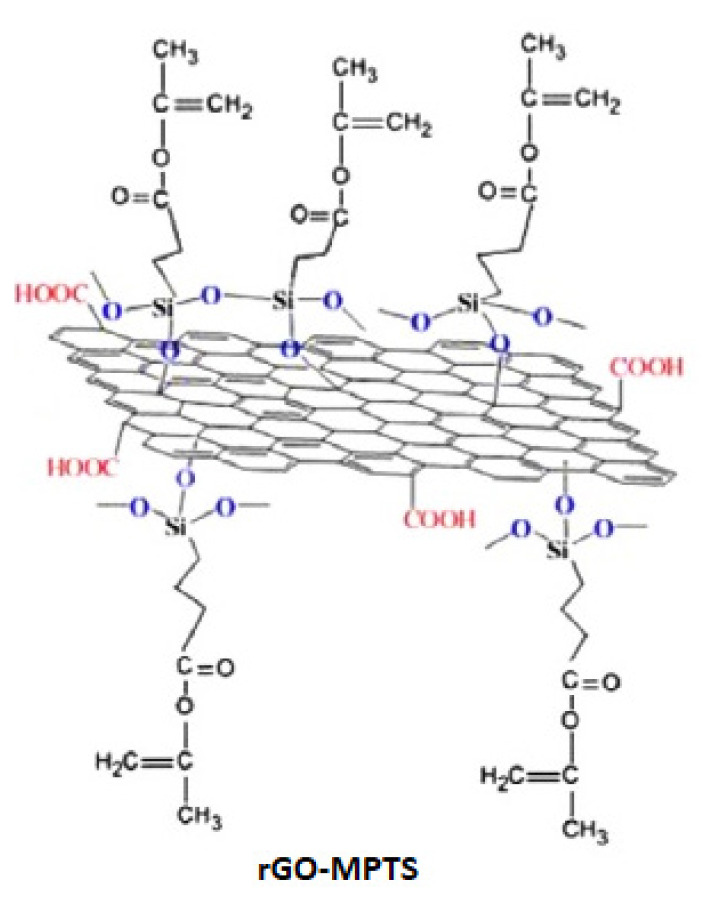
Structure of MPTS-rGO.

Nonimprinted (NIP) nanostructures were prepared in the polymerization environment with the same method without template molecules (folic acid). Synthesis steps and possible chemical interactions are shown in Figure 2. Figure 3 shows MPTS-rGO interactions.

### 2.2. Characterization of nanostructures

Synthesized nanostructures were characterized by Fourier transform infrared spectrometer (FTIR-ATR), Scanning Electron Microscopy-Energy Dispersive Spectrum (SEM-EDS) and Atomic Force Microscopy (AFM) methods.

#### 2.2.1. FTIR

The FTIR spectrum of synthesized nanomaterials was obtained using an FTIR spectrophotometer (FTIR 8000 Series, Shimadzu Japan). For this purpose, dried nanoparticles (2 mg) were homogeneously mixed with KBr (98 mg, IR Grade, Merck, Germany) and the FTIR spectrum was drawn in the range of 4000-450 cm^−^^1^ [26].

In this way, it was revealed whether nanostructures were successfully synthesized based on the specific bands of each functional group participating in the structure in the modification steps and imprinting processes of the material.

#### 2.2.2. SEM-EDS

After the nanostructures were dried in the study, it was powdered and SEM analysis was performed. This analysis provides information about the surface morphology of nanostructures

In addition to this, the presence of functional groups participating in the structure after modifications of synthesized nanostructures (GO, MPTS-rGO, FA-Imp-poly(MPTS-rGO-co-NAT) was investigated by SEM-EDS analysis.

#### 2.2.3. Atomic force microscopy (AFM)

AFM measurements were performed using the Digital Instruments-MMAFM Nanoscope IV atomic force microscope to learn about the morphology of synthesized nanostructures. For this purpose, nanostructures are distributed and dried on a glass surface of 1 cm × 1 cm in size. AFM measurements were made using tapping mode.

### 2.3. Electrochemical measurements

Imprinted and nonimprinted nanostructures were homogenized by 15 min of sonication with 5 mL of a 0.10 M acetic acid solution containing 0.5% chitosan. Chitosan is an adhesive substance used for the stabilization of electrodes modified with molecularly imprinted nanostructures. The 4 μL nanomaterial was dripped into SPCE’s carbon working electrode by using a micropipette and then dried at 50 °C. The NIP/SPCE sensor was prepared with the same procedure using NIP instead of MIP. To test the modification of the sensor surface by nanostructure, CV and DPV of the electrode modified with unmodified electrodes and nanostructures were taken.

#### 2.3.1. Preparation and use of the ferri-ferro solution

Potassium hexacyanoferrate (III) K3[Fe(CN)6] and potassium hexacyanoferrate (II) trihydrate used to prepare the ferri-ferro solution are affected by light, so the solution is carried out in a dark environment until it is transferred to the amber bottle. After weighing 745 mg KCI (0.1 M), 680.5 mg KH_2_PO_4_, 211.2 mg K_4_[Fe(CN)_6_],165 mg K_3_[Fe(CN)_6_] was weighed 5 mM (1:1) and dissolved in 100 mL ultra-pure water. Since the solution is affected by light during measurements, it is prepared fresh before each study.

#### 2.3.2. Determination of optimum binding conditions between folic acid and nanostructure (Imp-poly(MPTS-rGO-co-NAT) on the sensor surface

Differential pulse voltammetry (DPV) and circular voltammetry (CV) techniques were used to determine the binding conditions (pH, concentration) between folic acid and Imp-poly(MPTS-rGO-co-NAT nanostructures on the sensor surface. LOD and LOQ values were calculated based on the calibration graph obtained under optimum conditions.

#### 2.3.3. Selectivity studies

Sensor responses of the mixture obtained by mixing folic acid with dopamine and ascorbic acid, structural analogues of folic acid, were examined. Folic acid imprinting nanostructures (Imp-poly(MPTS-rGO-co-NAT) located on the sensor surface were evaluated in terms of folic acid determination selectivity. In this way, the selectivity of the developed Imp-poly(MPTS-rGO-co-NAT) nanostructures in the presence of structural analogues, which is the target molecule folic acid, was determined.

#### 2.3.4. Reusability

With nanosensors designed with Imp-poly (MPTS-rGO-co-NAT) nanostructures, measurements of folic acid of a certain concentration were performed and sensor responses were evaluated. For this purpose, a concentration of 10 ng/mL of folic acid was used in repeatability trials.

#### 2.3.5. Determination of folic acid in commercial human blood samples

Sensor responses were examined by adding folic acid in certain concentrations with the standard addition method to the commercially obtained blood sample (Human Blood/SIGMA ALDRICH BCR635-1EA) in terms of validation of the developed method.

## 3. Results and discussion

### 3.1. Characterization of nanostructures

#### 3.1.1. FTIR

**Figure 4:**
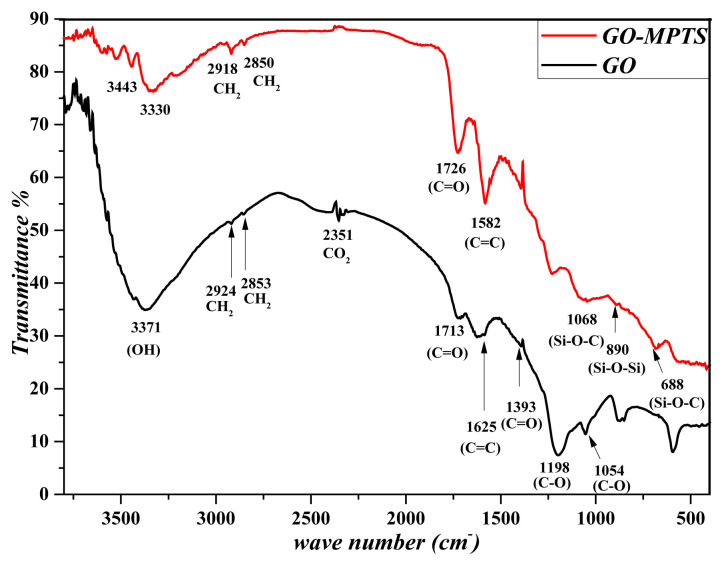
FTIR spectrums of GO and GO-MPTS nanostructures.

The FTIR spectrum of synthesized nanomaterials was obtained using an FTIR spectrophotometer (FTIR 8000 Series, Shimadzu Japan). For this purpose, dried nanoparticles (2 mg) were homogeneously mixed with KBr (98 mg, IR Grade, Merck, Germany) and the FTIR spectrum was drawn in range of 4000–450 cm^−^^1^ [26]. FTIR spectrums of GO and GO-MPTS nanostructures was given in Figure 4.

Carboxylic O-H stretching of GO in the FTIR spectrum appeared as a wide peak of 3371 cm^−^^1^, symmetrical and asymmetrical CH_2_ bands 2853 and 2924 cm^−^^1^, CO_2_-related peaks 2351 cm^−^^1^, C=O stretching vibrations at 1713 cm^−^^1^ and band hydroxyl band stretch vibration at 1393 cm^−^^1^. C=C skeletal vibrations are 1625 cm^−^^1^ and C-O-C epoxy in the epoxy group at 1054 and 1198 cm^−^^1^. Since these peaks are parallel with the literature, they indicate that GO has been successfully synthesized. As can also be understood from the FTIR spectrum, there are plenty of functional molecules (groups of carboxylic, hydroxyl, and carbonyl) on the surface of the synthesized GO.

When the FTIR spectrum of GO-MPTS is examined, the stretching vibrations of the bands -CH_2_ groups in 2918 and 2850 cm^−^^1^ in the spectrum, a strong absorption band at 1726 cm^−^^1^ refers to the stretch vibration of C=O groups. The band at 1582 cm^−^^1^ corresponds to vinyl groups of MPTS units on GO layers. The bands in spectrum 1068 and 688 cm^−^^1^ are caused by the stretching vibration of the Si-O-C bonds. Furthermore, the presence of bands in 1068 and 890 cm^−1^ related to (Si-O–O–C/Si-O–Si) is further proof that silanization has been successfully performed. However, the C=C stretching of the MPTS vinyl group coincides with the length band (1625 cm^−^^1^) of approximately 1580 cm^−^^1^ GO. In GO-MPTS, the shift of the C=O stress vibration to 1726 cm^−^^1^ is also evidence silanization.

#### 3.1.2. SEM-EDS

In order to make SEM measurements, nanostructures were distributed to the lamellar surface and then dried [27]. SEM analyses were performed using the Phillips XL-30S FEG device and the results are given below.

**Figure 5:**
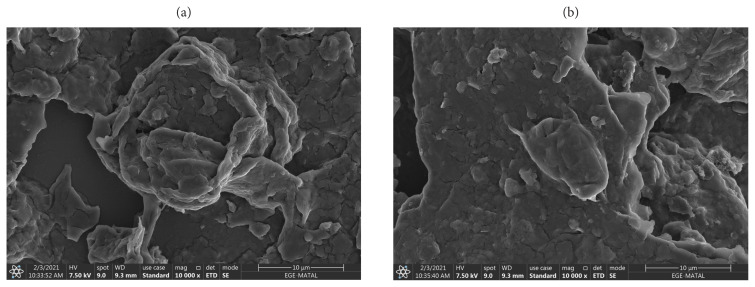
SEM images of (a) Folic acid Imprinted nanostructures and (b) nonimprint nanostructures.

As shown in Figure 5, it was observed that the layered graphene oxide structures seen in the SEM photo of imprint and nonimprint GO nanostructures has layered layer in accordance with the literature and overlapping GO structure in a scattered state.

The presence of functional groups added to the structure after modifications of synthesized nanostructures (GO, MPTS-rGO, and Imp-poly(MPTS-rGO-co-NAT) was investigated by SEM-EDS analysis. These data are given in Table 2. According to these results, it can be said that graphene oxide nanostructures have been successfully modified due to the fact that Si and N atoms in the functional groups of MPTS and NAT are also observed in the nanostructure.

#### 3.1.3. Atomic force microscopy (AFM)

**Figure 6:**
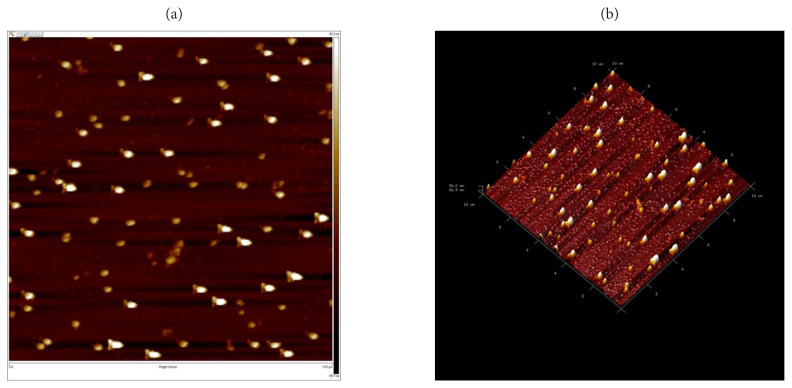
AFM images of (a) folic acid imprinted (b) nonimprinted nanostructures.

AFM measurements were performed using the Digital Instruments-MMAFM Nanoscope IV atomic force microscope to learn about the surface morphology of synthesized nanostructures. For this purpose, nanostructures are distributed and dried on a glass surface of 1 cm × 1 cm in size. AFM measurements were made using tapping mode. Figure 6 shows folic acid imprinted and nonimprint nanostructures obtained from AFM analysis.

### 3.2. Electrochemical measurements

PalmSens potentiostat (The Netherlands) was used for electrochemical analysis as part of nanosensor development. Differential pulse voltammetry (DPV) and circular voltammetry (CV) methods were used as electrochemical methods in the studies. In the applied methods, the ferri-ferro solution was prepared and used for electrochemical determination purposes, which allows learning about the charge of the surface by taking advantage of the reaction between the reduction and oxidation of iron in the solution. As the number of substances connected to the surface increases, it becomes difficult for the iron in the solution to reach the conductive part of the electrode. In light of this principle, the amount of connection on the surface can be calculated from the decreasing signal quantity due to decreased conductivity.

#### 3.2.1. Determination of optimum binding conditions between folic acid and nanostructure (Imp-poly(MPTS-rGO-co-NAT) on the nanosensor surface

**Figure 7:**
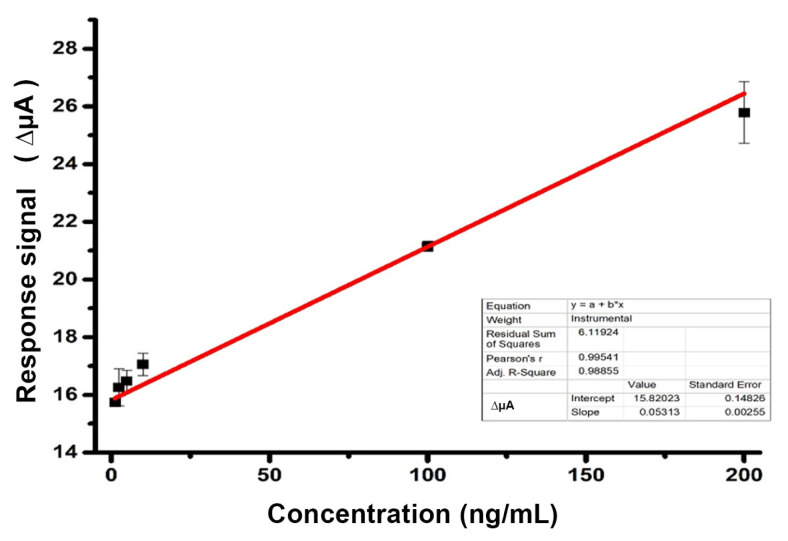
Calibration curve.

The details of the methods used for DPV and CV measurements and the electrograms given by empty electrodes are given below. Measurements in differential pulse voltage were performed at an application speed of 5.10^−2^ volts per second in the potential range of −0.4 to 0.6 volts. Measurements were made in 3 repetitions. DPV measurements were carried out at different folic acid concentrations (1.25, 2.5, 5, 10, 100, 200 ng/mL) in order to optimize folic acid binding time to the electrode surface connected to imprinted nanostructures. The calibration curve and properties of the nanosensor are given in Figure 7 and Table 3, respectively.

As we mentioned above, DPV technique was preferred for evaluating the analytical performance of the nanosensor. In response to the biosensor, the difference between the before (SPCE) and subsequent (SPCE/MIP-MPTS-rGO-co-NAT) and SPCE/MIP-MPTS-rGO co-NAT/FA DPV peak values in different concentrations was used (DμA). The calibration graph of the nanosensor was prepared in a buffer of 1.25, 2.5, 5, 10, 100, 200 ng/mL folic acid in a buffer of 10 mM Sodium phosphate (pH:7.4) and obtained by applying it to the nanosensor (Figure 7). Linearity between 1.25–200 ng/mL FA was observed according to the calibration graph obtained. This calibration chart was expressed with the correct equation y = 0.05313 x + 15.82 (R^2^ = 0.989). The slope standard deviation and % variation coefficient were calculated as 0.00255% and 2.29%, respectively ± as a result of 8 repeated measurements. After 8 measurements were taken using 10 ng/mL FA, the lowest detection limit (LOD) was calculated as 7.54 ng/mL with 3Sb/m formula, and the determination limit (LOQ) was calculated as 25.14 ng/mL with 10Sb/m formula. The analytical parameters of the nanosensor are summarized in Table 3.

#### 3.2.2. Electrochemical characterization of imprinted nanosensor

**Figure 8:**
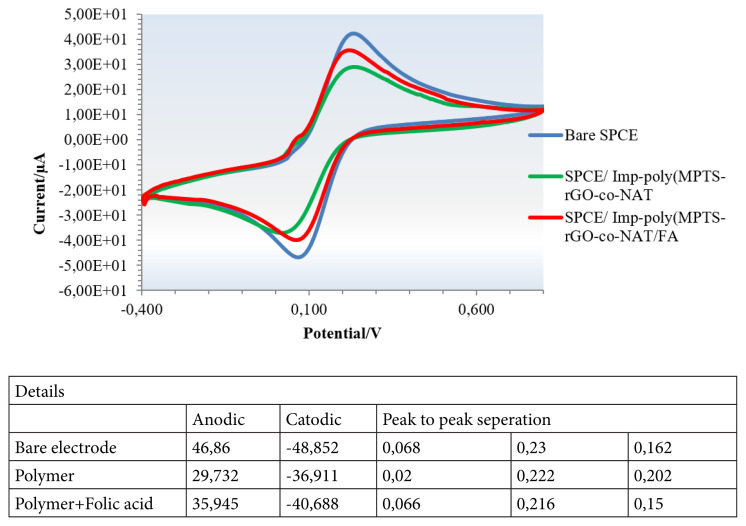
DPV diagrams. SPCE, SPCE/Imp-poly(MPTS-rGO-co-NAT, SPCE/Imp-poly(MPTS-rGO-co-NAT/FA. (50 mM sodium phosphate buffer, pH 7.4, 5.0 mM [Fe(CN)6]3−/4 and 0.1 M KCl )

**Figure 9:**
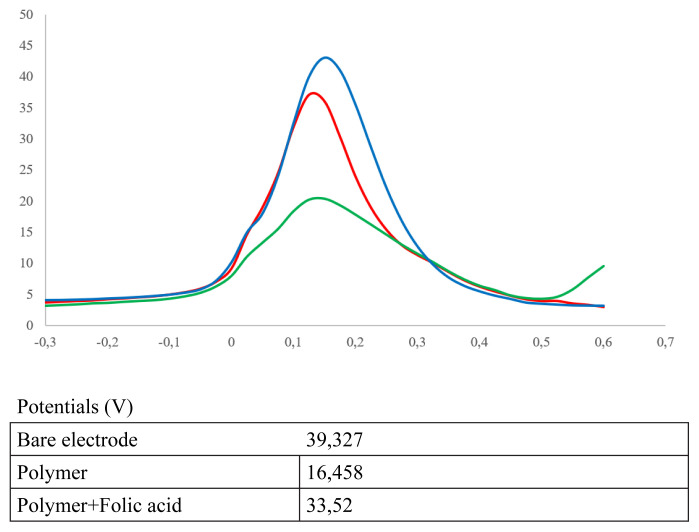
CV diagrams. SPCE, SPCE/Imp-poly(MPTS-rGO-co-NAT, SPCE/Imp-poly(MPTS-rGO-co-NAT/FA. (50 mM sodium phosphate buffer, pH 7.4, 5.0 mM [Fe(CN)6]3−/4 and 0.1 M KCl )

Sensor surfaces are designed as empty electrodes (SPCE), imprinted nanostructures (SPCE/Imp-poly(MPTS-rGO-co-NAT) and nanosensor surface and analyte interaction (SPCE/Imp-poly(MPTS-rGO-co-NAT/FA). Measurements were carried out using these surfaces. The anodic peak currents of the electrode surface were found as follows, respectively, 46,86 μA for SPCE, 29,732 for SPCE/Imp-poly(MPTS-rGO-co-NAT), and 35,945 μA for SPCE/MIP-MPTS-rGO-co-NAT/FA. Cathodic peaks are 48,852 μA for SPCE, 36,911 μA for SPCE/Imp-poly(MPTS-rGO-co-NAT), and 40,688 μA for SPCE/Imp-poly(MPTS-rGO-co-NAT/FA. When the vertical distances of the anodic and cathodic peaks are examined, it is seen that it is 0.162V for SPCE, 0.202V for SPCE/Imp-poly(MPTS-rGO-co-NAT, SPCE/Imp-poly(MPTS-rGO-co-NAT/FA 0.15V. Shifts in potentials and changes in peak potentials indicate that surface modifications have been successful (Figures 8 and 9).

#### 3.2.3. Selectivity studies

**Figure 10:**
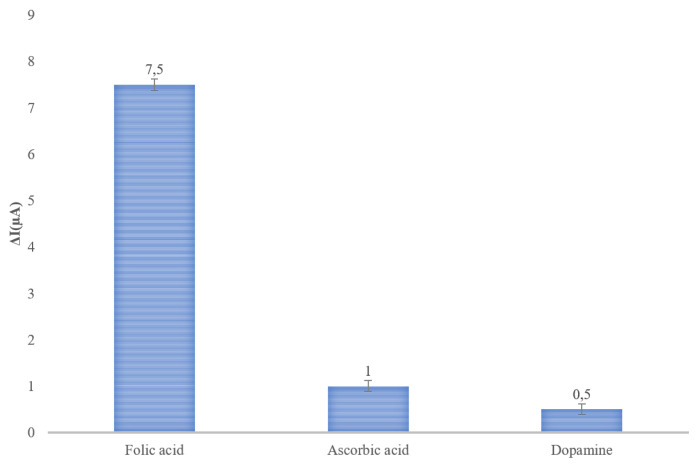
Selectivity of imprinted nanosensor.

In the selectivity studies, nanosensors were designed using sensor surfaces created with Imp-poly (MPTS-rGO-co-NAT) nanostructures. Sensor responses of these nanosensors as analytes with ascorbic acid, dopamine structural analogues of folic acid and folic acid were examined. To examine the selectivity of the imprinted nanosensor, it was prepared in 10 ng/mL concentration from all molecules (folic acid, ascorbic acid, and dopamine) and applied to the nanosensor system. Figure 10 gives a selectivity result.

As a result of DPV measurements, it was determined that the imprinted nanosensor system is approximately 7 times more selective to the folic acid molecule compared to its structural analogues. As a result, the nanosensor did not give a meaningful response to the structural anaologs of folic acid. This was interpreted as the result of the molecular imprinting technique performed, that the nanostructures used on the nanosensor surface recognize folic acid with high affinity.

#### 3.2.4. Reusability

**Figure 11:**
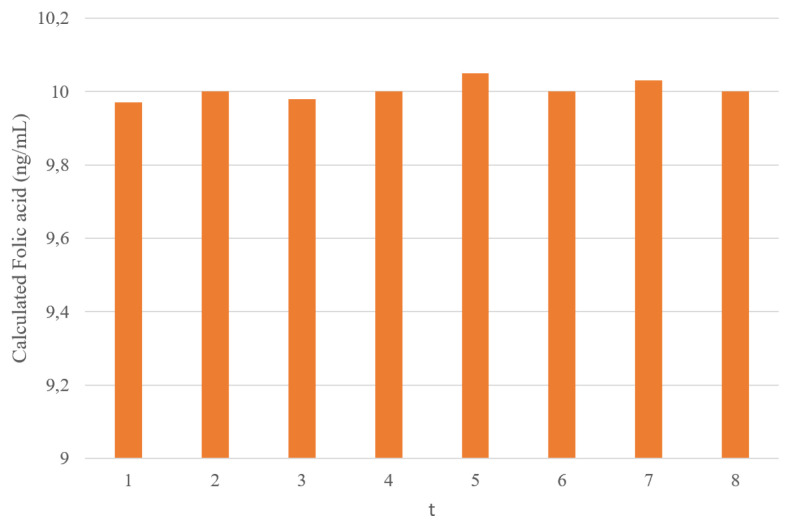
Reusability of imprinted nanosensor (V_polymer_: 4μL, V_FA_: 5μL, t repetitations: 8).

Reusability measurement results repeated 8 times in 10 ng/mL folic acid concentration were 9.97 ng/mL, 10.0 ng/mL, 9.98 ng/mL, 10 ng/mL, 10.05 ng/mL, 10 ng/mL, 10.03 ng/mL, 10 ng/mL. Considering that the actual value of 10 ng/mL is obtained quite close to the value, it can be said that the reusability of the developed imprinted nanobiosensor is high (Figure 11).

#### 3.2.5. Determination of folic acid in commercial human serum

Commercial synthetic human serum samples were used to test the developed nanosensor in real samples. Folic acid (10 and 20 ng/mL) was added to the human serum with the standard adding method and nanosensor measurements were performed with DPV technique. The similarity of the results indicates that the performance of the nanosensor was successful. (Table 4)

The low calculated % RSD value indicates that the precision of the method and the accuracy of the measured values are high [28]. It is seen that it can be used for clinical diagnosis in the human blood serum sample.

## 4. Conclusion

The normal concentration of FA in human serum is approximately 2–15 ng/mL [29]. Therefore, it is very important from a clinical perspective to determine the FA sensitively. Numerous methods such as spectrophotometric [30], thermogravimetry [31], and HPLC were applied in FA measurement [32].

Although these techniques are precise and accurate, they are expensive, complex, and time-consuming. In recent years, electrochemical methods have gained a lot of attention and have been widely used to detect and identify various biological compounds, organic molecules, and inorganic ions. Low cost, ease of application, high repeatability, rapid response, and low detection limit are some advantages that indicate the reason for the intense interest in electrochemical methods [33,34]. The literature data were examined, and the potentials of sensor systems developed for folic acid determination were investigated. Some of the studies carried out in this context are summarized below.

Arvand and Dehsaraei (2013) linked folic acid on the surface of the modified carbon electrode (AuNPs/CPE) with gold nanoparticles [35]. Without any complex and time-consuming steps, FA was determined in the human blood plasma sample. In the study, the FA limit of quantification was 6 × 10^−8^ and 8 × 10^−5^ mol L^−1^ (LOD) is 2.7 × 10^−8^. The MIP-based FA sensor is designed using a new monomer (2,4,6-trisacrylamido-1,3,5-triazine, TAT) technique on carbon. It is induced for direct electronic transmission into the carbon polymer. Only FA molecules trapped in the MIP cavity are oxidized on the surface of the MIP-fiber sensor. The cyclic voltammogram showed well-defined redox peaks. The method was found to be more specific and RSD = 1.3% to 0.20 ng/mL LOD was observed. The analysis was done in blood serum and pharmaceutical samples. The interference was found to be negligible during FA detection in biological samples [36]. In another study, Prasad et al., (2010), an electrochemical sensor was developed using a preprepared sol-gel coated pen graphite (2D class) electrode with molecularly imprinted polymer for selective and quantitative recognition of folic acid [37]. As a result of electrochemical studies performed with molecularly imprinted polymer synthesized using functional monomer to triaminotriazine, the linear range 0.007–0.156 μg/mL determination limit was determined as 0.002 μg/mL. Benvidi et al. (2015) produced glassy carbon electrode (GCE) modified with reduced graphene oxide (rGO), gold nanoparticles, and 2-(3,4-dihydroxy phenyl) benzothiazole (DHB), gold nanoparticles by electrochemical deposition method on rGO/GCE. This sensor was simultaneously applied to the determination of levodopa (LD), uric acid (UA), and folic acid (FA). The developed electrode determined the determination of LD, UA, and FA in some standard samples (e.g., Madopar tablet, urine, and human blood serum) by the standard adding method [38]. Chekin et al. (2016) prepared and characterized a matrix based on molybdenum disulfide reduced graphene oxide hybrid (MoS2-rGO). The modification of the glassy carbon electrode (GCE) with MoS_2_-rGO (MG) was carried out by drip and drying method on the electrode surface. In experiments conducted in the presence of various types of interventions, the 10 nM determination limit was determined, and the linear range was 0.01 μM–100 μM [39]. In their study He et al. (2016), they modified the sensor surface by electrophoretic accumulation (EPD) on golden electrodes, rGO adsorption, and the functionalization of rGO with folic acid. Using this method, a determination limit of 1 pM is obtained [40]. The method also ensured the detection of folic acid in serum. Rajabi et al. (2017) synthesized with a new two-phase (organic-water) synthesis method of poly (ortho-metoxyanilin) nanostructures (POMANS) for the determination of folic acid (FA) and uric acid (UA), and this polymer was used to create a modified multi-walled carbon nanotube graphite paste electrode (POMANS-MWCNT/GPE). The assignment limit for UA and FA is 0.157 and 0.113 μM respectively [41]. Kuceki et al. (2018) aim to develop a graphite/limited access molecularly suppressed poly (metacritical acid)/SiO2 composite-based voltammetric sensor for folic acid determination in pharmaceutical formulations. Experiments with graphite cake electrodes prepared with limited access hybrid molecular imprinted polymer (RAM-MIP) resulted in an increase in cathodic peak current for FA. Since the electrical conductivity of RAM-MIP is very poor, it has been found that the optimum content is very low (%3, w/w) in the composite. Under conditions optimized using square wave voltammetry, linear ranges between 0.72 μg L^−^^ 1^ (1.63 nmol L^−^^1^) and 5.0 to 100.0 μg L^−^^ 1^ (0.01 to 0.23 μmol L^−^^ 1^) were obtained [42].

The FA is a possible tumour marker for cancer screening. It has been accepted that the concentration of FA in the blood of cancer patients is lower than that of healthy individuals [43]. However, for the determination of this biological token at a low concentration (range of picomolar), it requires the use of ultra-precise detection methods. In addition, folic acid determination is important in diseases such as cardiovascular diseases, neural tube defects, cleft lip and palate, late pregnancy complications, neurodegenerative and psychiatric disorders. Therefore, from a clinical point of view, it is very important that the FA is appointed sensitively. However, only two of the sensor systems developed for FA determination in the above literature summaries offer sufficient precision for the real-time determination of the FA. Numerous methods have also been developed for FA determination, such as spectrophotometry [30], thermogratrometry [31], and high-performance liquid chromatography [44]. Although these techniques are precise and highly repeatable, they are expensive, complex, and time-consuming. In the clinic, the chemiluminescence immunoassay method (CL-IA) is currently used for folic acid determination. In the CL method, marking is done with a substance that gives chemiluminescence. In this method, analysis is mandatory on a single device and, accordingly, a single kit. They are also expensive compared to open systems. Since the CL reagent is not specific to single analytics, its selectivity is low. Accordingly, detector responses resulting from measurement may be misleading [45]. CL emission densities are affected by a variety of environmental factors, such as temperature, solvent, ionic force, pH, and other types in the system. Also, since the emission intensity from the CL reaction varies depending on the time (a light increase consisting of a signal increase after the reagent is mixed passes through the maximum and falls to the baseline), the time profile against CL emissions varies from one compound to another. Therefore, care should be taken to detect the difference in current occurring at times defined as references. In many CL systems, there is a low level of background emissions in the absence of an analyte. Therefore, in streaming systems, CL signals increase in proportion to analyte concentration when the mixture of analyte and reagent(s) passes through the detector cell and appear as overlapping sharp peaks when displayed by the time window. Due to the fact that CL emissions are measured only from this small-time profile, nonlinear graph can be obtained when reactions with complex kinetics are graphed against analyte concentration [46].

In this study, it is aimed to develop an alternative sensor system to the chemiluminescence immunoassay method used for folic acid determination. The folic acid-imprinted nanomaterials recommended in the study were synthesized for the first time for this purpose. Some characteristics of the developed nanosensor; detection limit (LOD) was 7.54 ng/mL and determination limit (LOQ) were 25.14 ng/mL. FA analytical (10 and 20 ng/mL) was added to commercial synthetic serum samples by standard adding method and RSD values of 0.092% and 0.734% were found in DPV technique and measurements respectively.

The design, synthesis, and modification methods of the imprinted nanostructure used in the study are unique. This nanostructure is also unique in terms of its use in the nanosensor developed for the determination of folic acid. It has been determined that the nanosensor developed for the determination of folic acid within the scope of the study has high selectivity, requires a small sample volume, does not require pretreatment, has a low detection limit and cost, and can respond quickly.

## Figures and Tables

**Table 1:** Polymerization conditions and recipe of polymerization.

Template/functional monomer (FA/NAT) concentration ratio	0.5
Functional monomer/sylanize graphene oxide (NAT/MPTS-rGO) ratio	5 mmolg^−1^
Functional monomer/crosslinker (NAT/EGDMA) mol ratio	1/10
Amount of initiator (KPS)	0.020 g

**Table 2:** Elemental analysis results obtained with SEM-EDS technique.

Nanostructure	GO 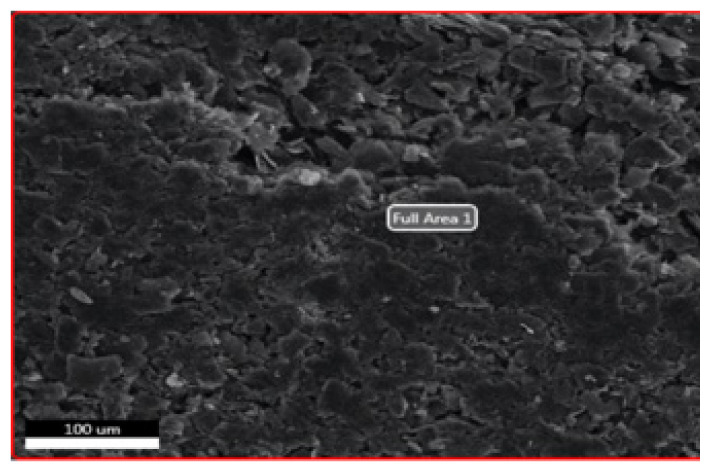		MPTS-rGO 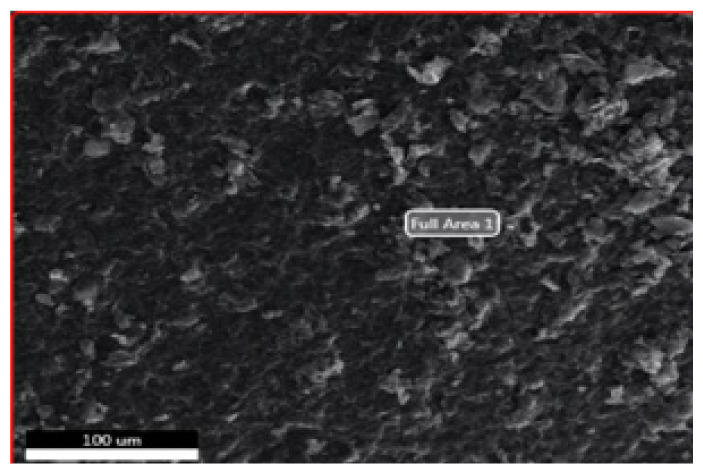		FA-Imp-poly(MPTS-rGO-co-NAT) 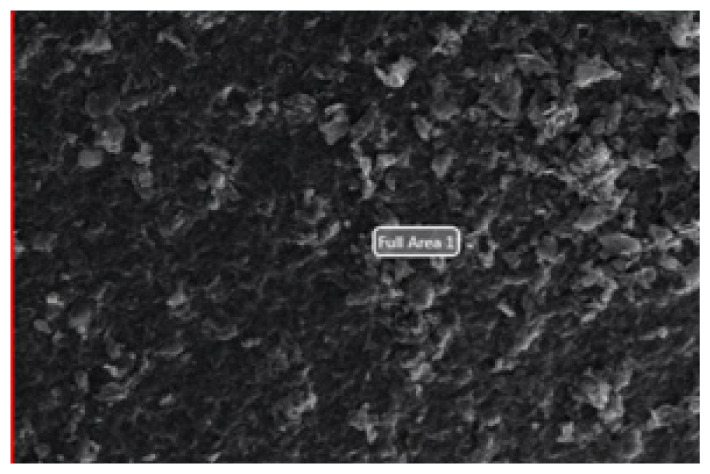	
Element	Weight %	Atomic %	Weight %	Atomic %	Weight %	Atomic %
C K	83.33	86.96	62.76	71.7	61.45	70.15
N K	-	-	-	-	0.7	0.36
O K	16.67	13.04	28.76	24.41	30.69	26.3
SiK	-	-	8.48	2.89	7.16	3.19

**Table 3:** Properties of the nanosensor.

Graphic equation	y = 0.05313 x + 15.82
R^2^	0.9886
y intercept (intersection)	15.82023
y intercept standard deviation	0.14826
Slope (m)	0.05313
Slope standard deviation	0.00255
LOD: 3Sb/m (limit of detection)	7.543 ng/mL
C.V. (% Variation coefficient)	2.291
LOQ: 10Sb/m (limit of quantification)	25,142 ng/mL

**Table 4:** Folic acid levels measured by standard added method.

Added Folic acid, ng/mL	Found in folic acid, ng/mL	Recovery	%RSD
10	9.987 ± 0.009	99.87 %	0.092
20	20.21 ± 0.148	101.05 %	0.734
